# Clinical significance of progastrin-releasing peptide, neuron-specific enolase, chromogranin a, and squamous cell cancer antigen in pulmonary neuroendocrine tumors

**DOI:** 10.3906/sag-1810-147

**Published:** 2019-06-18

**Authors:** Nuri TUTAR, Nur Aleyna YETKİN, Cevat YAZICI, Ömer ÖNAL, Olgun KONTAŞ, Fahrettin KELEŞTEMUR

**Affiliations:** 1 Department of Pulmonary Medicine, Faculty of Medicine, Erciyes University, Kayseri Turkey; 2 Department of Pulmonary Medicine, Faculty of Medicine, Health Sciences University, Kayseri Turkey; 3 Department of Biochemistry, Faculty of Medicine, Erciyes University, Kayseri Turkey; 4 Department of Thoracic Surgery, Faculty of Medicine, Erciyes University, Kayseri Turkey; 5 Department of Pathology, Faculty of Medicine, Erciyes University, Kayseri Turkey; 6 Turkish Health Institutes, Ankara Turkey

**Keywords:** Neuroendocrine tumor, lung neoplasm, markers, carcinoid

## Abstract

**Background/aim:**

It is not always easy to diagnose pulmonary neuroendocrine tumors (PNETs). The aim of the present study is to make a differential diagnosis by studying the same markers in patients with non-small-cell lung carcinoma (NSCLC), patients with benign lung disease (chronic obstructive pulmonary disease and pneumonia), and healthy volunteers to determine the roles of these markers in pulmonary neuroendocrine tumor diagnosis and to identify their power.

**Materials and methods:**

A total of 100 participants including 23 PNET patients and 28 NSCLC patients who were pathologically diagnosed but not yet treated, 25 participants with benign disease, and 24 healthy volunteers were included in this cross-sectional study.

**Results:**

No significant difference was found between the chromogranin A (CgA) and squamous cell carcinoma antigen 1 (SCCA1) values among the groups (PNET, NSCLC, benign, healthy volunteers), but the difference in progesterone-releasing peptide (ProGRP), neuron-specific enolase (NSE), and adjusted NSE was statistically significant (P values were respectively ProGRP, P = 0.006; NSE, P = 0.015; NSE adjusted, P = 0.09). In a comparison of the PNET and NSCLC groups, having a ProGRP value higher than 84.6 pg/mL revealed PNET with 60.9% sensitivity and 89.3% specificity (P = 0.001).

**Conclusion:**

The ProGRP value is the only indicator that distinguishes the PNET group from the other 3 groups.

## 1. Introduction

Pulmonary neuroendocrine tumors (PNETs) constitute approximately 20% of lung cancers [1–3]. According to the European Neuroendocrine Tumor Society expert consensus report, neuroendocrine tumors are classified as typical carcinoid (TC), atypical carcinoid (AC), small-cell lung cancer (SCLC), and large cell neuroendocrine tumor (LCNT) [4]. Approximately 90% of tumors with endobronchial involvement are symptomatic and may present with hemoptysis, cough, recurrent pulmonary infections, and unilateral wheezing. However, 1/2 to 1/5 of PNET patients are asymptomatic [5–8]. The diagnosis of these patients is often coincidental with the detection of a lesion via an imaging method applied for another reason. However, with small biopsy specimens, carcinoid tumors may be mistakenly diagnosed as SCLC, whereas LCNT may be misdiagnosed as poorly differentiated adenocarcinoma, squamous cell carcinoma, or basaloid carcinoma. In this context, the question is whether the diagnosis of PNET patients can be supported by using some additional tests. For this purpose, attempts have been made to develop various immunohistochemical and biochemical markers. 

The most commonly used immunohistochemical markers for neuroendocrine tumors are chromogranin, CD56, synaptophysin, and Ki-67 [8]. Progastrin-releasing peptide (ProGRP), used as a biochemical marker, is the precursor of GRP and is used as a tumor marker instead of nonstable GRP because its half-life is very short. ProGRP is a more frequently studied marker in SCLC; the sensitivities are in the range of 47%–86% and the specificity is over 90% when the threshold value is from 33.8 to 53 pg/mL [9–14]. Even if the result is >100 pg/mL, it has been emphasized that a neuroendocrine origin or small-cell component must be searched for even if the pathological diagnosis is NSCLC [15]. Neuron-specific enolase (NSE) is the neuroendocrine-specific isoenzyme of enolase. It plays a role in aerobic glycolysis and is found in many neural and neuroendocrine tumors [16]. Its sensitivity is 20%–81% in SCLC; when found positive, a better prognosis is suspected [16,17]. It has also been shown to be elevated in atypical carcinoids along with SCLC [18]. Chromogranins are acidic secretory proteins released from neuronal or neuroendocrine cells [16]. They were found to be high in 75% of 20 cases of pulmonary carcinoid tumors in which plasma chromogranin A (CgA) levels were studied [19]. In a study examining the CgA and NSE levels in SCLC, chromogranin sensitivity was found to be higher (61% and 57%, respectively) [20]. Squamous cell carcinoma antigen 1 (SCCA1) was originally demonstrated in squamous cell carcinoma of the cervix; elevated serum levels suggest lung squamous cell carcinoma when evaluated with masses in the lungs. Taking a threshold value of 1.5 ng/mL, only 7.5% of patients with SCLC were found to be positive [21]. For this reason, negative SCCA1 detection with other positive serum markers, especially in neuroendocrine lung cancer, increases the likelihood of diagnosis. The authors noted that sensitivity was 79.5% and specificity was 99.6% for SCLC in the use of SCCA1 (when used as an exclusion criterion) in combination with NSE and ProGRP [16,22]. Although these are the current available data, as of yet no prospective studies have evaluated these 4 tests in PNET cases. 

The primary aim of this study is to determine the sensitivity, specificity, negative predictive value, and positive predictive value of ProGRP, NSE, CgA, and SCCA1 obtained from peripheral venous blood samples in pathologically diagnosed PNET cases. In order to determine the role of these markers in PNET diagnosis and their power in the differential diagnosis, we aimed to make the differential diagnosis by studying the same markers in patients with NSCLC, patients with benign lung disease (chronic obstructive pulmonary disease and pneumonia), and healthy volunteers.

## 2. Materials and methods

### 2.1. Patient and control groups

The project was approved by the ethics committee of Erciyes University on 26.09.2014 with number 2014/528. Enrollment of participants in the project began on 15 June 2015; within 20 months of this date, all participants were enrolled in the study. 

Patients who were diagnosed with PNET (TC, AC, LCNT, SCLC) or NSCLC between the ages of 18 and 80 and had not received any treatment (surgery, chemotherapy, or radiotherapy) were included in the study. Those with benign pulmonary disease (chronic obstructive pulmonary disease [COPD], pneumonia) and healthy volunteers were also included. Active hepatic disease (active hepatitis B, active hepatitis C, and cirrhosis), a glomerular filtration rate of <30 mL/min, and regular proton pump inhibitor intake within the last month were the exclusion criteria for this study.

A total of 100 participants including 23 PNET and 28 NSCLC patients, 25 participants with benign disease, and 24 healthy volunteers were included in this cross-sectional study. An informed consent form was obtained from all of the participants.

### 2.2. Staging of PNET and NSCLC groups

Routinely used F18-labeled fluorodeoxyglucose (FDG) positron emission tomography-computed tomography (PET-CT) was used in the pretreatment staging of PNET and NSCLC cases. Staging of these cases was performed according to the 8th staging system [23]. In the PNET group, cases of SCLC were staged as limited disease or extensive disease [24]. 

### 2.3. Biochemical analysis

ProGRP, NSE, CgA, and SCC/A1 were studied in blood samples obtained from all patients and healthy control subjects. Serum/plasma samples obtained from venous blood samples were stored at –70 °C in the deep-freeze room of the Erciyes University Medical Faculty’s Chest Diseases Clinic, and all of the samples were studied when the target number of participants was obtained. 

Serum NSE and ProGRP levels were studied in Erciyes University’s Central Biochemistry Laboratory with a Roche Cobas E601 device (Roche Diagnostics GmbH, Mannheim, Germany) and commercial kit (ProGRP Catalog No. 06505961190, NSE Catalog No. 12133113 122, Roche Diagnostics GmbH). In order to monitor the quality of the ProGRP measurements, the control sera obtained from the manufacturer where the measurement was made were also measured with the same kit and device. The 2 measurement values of the ProGRP control 1 serum with a measurement target range of 35.0–60.8 pg/mL were found to be 43.94 and 44.87 pg/mL, and the 2 measurement values of the ProGRP control 2 serum with a measurement target range of 507.0–883.0 pg/mL were found to be 717.4 and 722.7 pg/mL. Since these control sera were used to measure the desired target range, the ProGRP assays were deemed to be accurate.

Since hemolysis causes false increments in NSE values, hemolysis-free NSE values were also calculated in accordance with the literature and the results were given as adjusted NSE [25]. 

Serum CgA and serum SCCA1 levels were measured in accordance with the manufacturer’s manual with commercial ELISA kits numbered MBS704285 and MBS721625 (MyBioSource, Inc., San Diego, CA, USA), respectively. 

### 2.4. Statistical methods

SPSS 22.0 (IBM Corp., Armonk, NY, USA) was used for statistical analysis. The distribution of continuous variables was tested with the one-sample Kolmogorov–Smirnov test, and the data are shown as mean ± standard deviation or median and minimum–maximum intervals. CgA, ProGRP, NSE, adjusted NSE, and SCCA1 data were given using medians and 25%–75% ranges. Categorical variables were reported as frequency and group percentiles. The receiver operating characteristic (ROC) curves were obtained by individual analysis of the serum markers of patients and control groups using the easyROC [26] and medCalc (1993–2017 MedCalc Software bvba, Ostend, Belgium) programs and the area under the curve was calculated. The threshold value was calculated using the Youden index and the sensitivity, specificity, positive predictive value, and negative predictive value were found with the medCalc program according to this value. All P-values were two-tailed and P < 0.05 was considered significant. In the power analysis performed before the study, a value of 0.40 was used considering the high effect size proposed by Cohen [27]. The number of units for each variable determined as 90% power and 5% for type 1 error was 24 for each group and at least 96 in total. The power for the relevant variables after the study was calculated as follows with type 1 error of 5%: ProGRP, 96.1%; NSE, 96.6%; CgA, 22.8%; SCCA1, 4.9%; adjusted NSE, 98.4%.

## 3. Results

A total of 100 participants including 23 PNET patients (23%) (7 TC, 2 AC, 14 SCLC), 28 NSCLC patients (28%) (15 adenocarcinoma, 13 squamous cell carcinoma), 25 participants with benign disease (25%) (8 pneumonia, 17 COPD patients), and 24 healthy volunteers (24%) were included in this study. When the age differences between the groups were examined, it was found that the difference was statistically significant; the reason for this significance was that the average age of healthy participants was lower than that of the patients in the NSCLC and benign groups (P < 0.05). While males were dominant in the PNET and NSCLC groups, females were dominant in the healthy volunteers (P < 0.05). Demographic findings related to the patients are shown in Table 1.

**Table 1:** Disease groups and demographic data.

	CgA	ProGRP	NSE	NSE adjusted	SCCA1
PNET–HealthyThreshold value AUC PSensitivity (%)Specificity (%)PPV (%)NPV (%)	1333.30.582NS39.187.575.060.0	95.20.7480.00160.910010072.7	40.90.6830.02747.895.891.765.7	12.70.7280.00360.987.582.470.0	49.30.599NS37.587.075.057.1
PNET–BenignThreshold value AUC PSensitivity (%)Specificity (%)PPV (%)NPV (%)	1564.20.519NS56.560.056.560.0	87.30.7340.00360.992.087.571.9	51.10.584NS47.810010067.6	25.40.6890.01847.810010067.6	59.90.546NS40.091.383.358.3
PNET–NSCLCThreshold value AUC PSensitivity (%)Specificity (%)PPV (%)NPV (%)	1404.90.547NS60.957.153.864.0	84.60.6990.01660.989.382.473.5	54.00.530NS47.878.664.764.7	35.30.586NS47.882.168.765.7	23.30.502NS71.443.560.655.6

*: Given as mean ± standard deviation. #: The age differences were statistically significant; the reason for this significance was the fact that the average age of healthy participants was lower than that of the patients in the NSCLC and benign groups (P < 0.05). &: The difference was statistically significant (P < 0.05). π: The difference was not statistically significant (P > 0.05).

When carcinoid tumors (typical and atypical) were staged, it was found that 3 patients (33.3%) were at stage 1a3 and 2 patients (22.2%) were at 2b. In small-cell lung cancer patients, 13 patients (92.9%) had extensive disease and 1 patient (7.1%) had limited disease. The stages of the patients are given in Table 2.

**Table 2:** Staging of malignant cases.

	n (%)
PNET Tumor type Typical carcinoid Atypical carcinoid Small cell lung cancer Smokingπ Active or ex-smoker Nonsmoker Male/female& Age*	237 (30.4)2 (8.7)14 (60.9)21 (91.3)2 (8.7)19/456.9 ± 12.9
NSCLC Tumor type Adenocarcinoma Squamous cell carcinomaSmokingπ Active or ex-smoker NonsmokerMale/female& Age*	2815 (53.6)13 (46.4)24 (85.7)4 (14.3)25/361.5 ± 10.9
Benign Subdisease Pneumonia COPDSmokingπ Active or ex-smoker Nonsmoker Female/male&Age*	258 (32)17 (68)18 (72)7 (28)12/1358.9 ± 15.0
Healthy controlSmokingπ Active or ex-smoker NonsmokerMale/female& Age*#	2417 (70.8)7 (29.2)7/1748.5 ± 9.3

**Figure 1:**
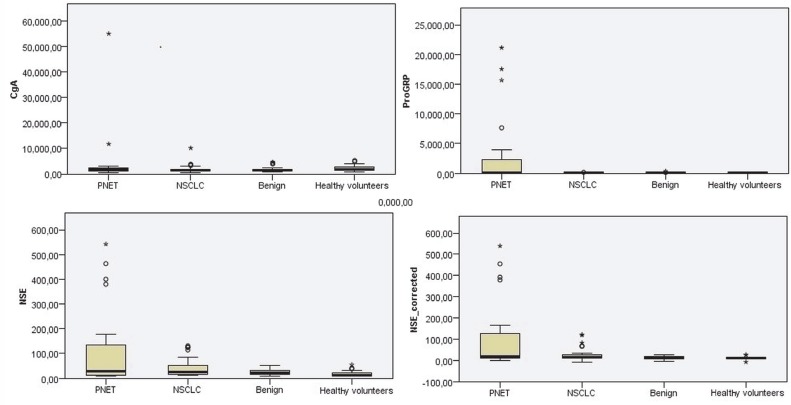
Comparison of CgA (chromogranin A, ng/mL), ProGRP (progastrin-releasing peptide, pg/mL), NSE (neuron-specific enolase, ng/mL), and NSE-adjusted values in patient groups and healthy volunteers.

There was no significant difference among the 4 groups (PNET/ NSCLC/benign/healthy volunteers) in terms of CgA and SCCA1 values, but the difference in the ProGRP, NSE, and adjusted NSE values was found to be statistically significant (ProGRP, P = 0.006; NSE, P = 0.015; adjusted NSE, P = 0.09). Table 3 gives the numerical values of these parameters in detail. In addition, the box-plot curves of these values are given in Figure 1.

**Table 3:** Progastrin-releasing peptide (ProGRP), neuron-specific enolase (NSE), NSE adjusted, chromogranin A (CgA), and squamous cell carcinoma antigen 1 (SCCA1) values in the pulmonary neuroendocrine tumors (PNET), non-small-cell lung carcinoma (NSCLC), benign, and healthy groups.

	n (%)
PNET (n = 23)Carcinoid tumors (n = 9) 1a2 1a3 1b 2a 2b 4a Small cell lung cancer (n = 14)Limited stage diseaseExtensive stage disease	1 (11.1)3 (33.3)1 (11.1)1 (11.1)2 (22.2)1 (11.1)1 (7.1)13 (92.9)
NSCLC (n = 28) 1a2 1a3 1b 2b 3a 3b 3c 4a 4b	1 (3.6)1 (3.6)1 (3.6)1 (3.6)2 (7.1)5 (17.9)2 (7.1)8 (28.6)7 (25)

Values are given as median (25%–75%). *: The difference was due to the high ProGRP levels in patients with PNET. #: The difference was due to the high NSE levels in patients with PNET. &: The difference was due to the high NSE adjusted levels in patients with PNET.

The reason for the significance of ProGRP among the 4 groups was the significant difference among all groups compared to PNET (PNET–NSCLC, PNET–benign, and PNET–healthy, P = 0.042, P = 0.002, and P = 0.002, respectively). The difference in these 4 groups of NSE was due to the significant difference between the PNET–healthy, NSCLC–benign, and benign–healthy groups (P = 0.005, P = 0.005, P = 0.03, respectively). When the adjusted NSE values were examined, it was determined that the significant differences between the PNET–healthy, PNET–benign, and NSCLC–healthy groups affected the total analysis (P = 0.003, P = 0.023, P = 0.018, respectively).

**Figure 2:**
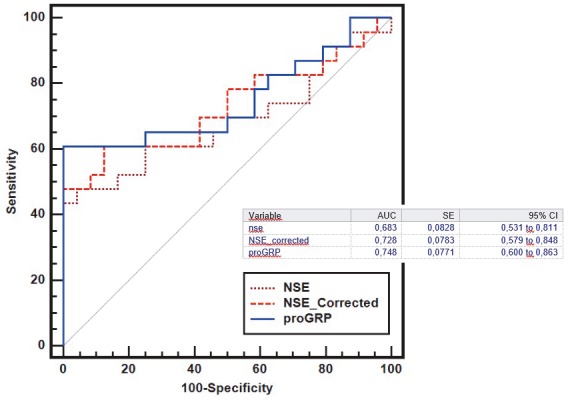
Multiple ROC analysis of ProGRP (progastrin-releasing peptide), NSE (neuron-specific enolase), and adjusted NSE values, which are significant in distinguishing the healthy group from the pulmonary neuroendocrine tumor (PNET) group; P > 0.05 for all values. AUC, Area under the curve; SE, standard error of the AUC; ProGRP, progastrin-releasing peptide; NSE, neuron-specific enolase. Statistics using two-tailed Z-test.

A comparison of PNET and other groups with ROC analysis in terms of CgA, ProGRP, NSE, adjusted NSE, and SCCA1 is given in Table 4. It was found that NSE and adjusted NSE values higher than 40.9 ng/mL and 12.9 ng/mL, respectively, for both groups revealed PNET with 47.8% and 60.9% sensitivity and 87.5% and 87% specificity, respectively (P < 0.05). When multiple ROC analyses were conducted on the ProGRP, NSE, and adjusted NSE values, which were significant in the comparison of the PNET and healthy groups, no parametric superiority to another was detected (P > 0.05) (Figure 2). In the comparison of the PNET and NSCLC groups, it was found that a ProGRP value higher than 84.6 ng/mL revealed PNET with 60.9% sensitivity and 89.3% specificity (P = 0.001).

**Table 4:** Comparisons of pulmonary neuroendocrine tumors (PNET) and other groups with ROC analysis in terms of progastrin-releasing peptide (ProGRP), neuron-specific enolase (NSE), adjusted NSE, chromogranin A (CgA), and squamous cell carcinoma antigen 1 (SCCA1) values.

	PNET (n = 23)	NSCLC (n = 28)	Benign (n = 25)	Healthy (n = 24)	P
CgA (ng/mL)	1644.5 (946.9–1644.5)	1363.4 (1068.1–1916.0)	1554.4 (1034.7–1900.8)	1675.8 (1419.2–2786.5)	>0.05
ProGRP (pg/mL)	151.8 (39.8–2563.0)	56.8 (37.9–78.5)	45.9 (34.5–59.)	47.9 (32.9–62.6)	0.006*
NSE (ng/mL)	29.0 (12.0–147.4)	24.3 (15.8–52.8)	22.0 (15.5–33.0)	13.2 (11.9–22.5)	0.015#
NSE adjusted (ng/mL)	17.1 (9.6–135.1)	13.8 (9.9–28.6)	11.3 (7.3–17.8)	9.8 (8.6–11.6)	0.009&
SCCA1 (pg/mL)	27.8 (19.6–46.06)	32.9 (21.6–43.4)	34.5 (16.9–75.3)	36.8 (24.7–58.4)	>0.05

AUC: Area under the curve; PPV: positive predictive value; NPV: negative predictive value; NS: not significant. The ProGRP and SCCA1 threshold values are given in pg/mL, whereas the CgA and adjusted NSE threshold values are given in ng/mL.

The hypothesis that SCCA1 squamous cell lung cancer can be used to differentiate the other NSCLC and PNET groups was also studied. However, no significant differences were found in distinguishing squamous cell lung cancer patients from other adenocarcinoma patients or distinguishing them from PNET patients (in the PNET group, 27.8 pg/mL (19.6–46.1); in adenocarcinoma patients, 32.1 pg/mL (16.3–43.8); in squamous cell carcinoma cases, 33.8 pg/mL (21.6–44.3); values are given as the median value (25%–75%, P > 0.05)). However, ROC analysis for SCCA1 values between the PNET and squamous cell carcinoma and the squamous cell carcinoma and adenocarcinoma groups did not yield a significant result (P > 0.05).

## 4. Discussion

In this study, the ProGRP value was found to be the only marker that distinguished the PNET group from the other 3 groups. The threshold value was found to be 95.2 pg/mL for the ProGRP value in the comparison of the PNET group and the healthy group and 87.3 pg/mL in the comparison of the benign group and the PNET group. In the comparison of the PNET and NSCLC groups in our study, ProGRP and adjusted NSE values higher than 84.6 pg/mL revealed PNET with 60.9% sensitivity and 89.3% specificity. In a study investigating diagnostic and prognostic criteria in NSCLC, ProGRP with a specificity of 95% and a sensitivity of 78.4% was reported [11]. In a similar study conducted by Molina et al., ProGRP was shown to have a sensitivity of 60%–70% for detecting limited SCLC and 75%–90% for detecting extensive SCLC [22]. Another study showed that the ProGRP level was found to be higher than those of NSE and chromogranin A in a comparison of SCLC and NSCLC [9]. These values are similar to those in our study. In addition, although age and sex were different between groups in the present study, Korse et al. showed a weak association of ProGRP with age and no association with sex in 282 neuroendocrine tumor patients and 297 healthy volunteers [28].

In this cross-sectional study, both NSE (threshold value of 40.9 ng/mL) and adjusted NSE (threshold value of 13.9 ng/mL) were significant in distinguishing PNET from the healthy group, whereas only adjusted NSE (threshold value of 25.4 ng/mL) was significant in a comparison of the PNET and benign groups. In a study in which CYFRA21.1, carcinoembryonic antigen, SCCA1, and NSE were studied in SCLC, the sensitivity of NSE was significantly higher than that of the other markers (sensitivity 81.2%) [22]. However, the sensitivities determined in other studies ranged from 43% to 52% [16]. In another study, ProGRP was found to be superior to NSE in distinguishing SCLC from benign disease and NSCLC [29]. In our study, the sensitivity of both NSE and adjusted NSE to distinguish between SCLC and NSCLC was 47.8%, but this difference was not statistically significant. In addition, although NSE can provide important information in the diagnosis and follow-up of SCLC, evaluation of serum NSE levels should be done with caution. NSE caused by fragmented red blood cells and thrombocytes in hemolyzed samples may lead to false positive evaluation [30]. We obtained adjusted NSE values in our study using a hemolytic index to be compatible with the study of Verfaillie et al. to avoid this mistake [25]. However, when the methodologies of studies performed with lung cancer cases were examined, the use of adjusted NSE values was not clearly understood.

Several studies tested the utility of CgA in the diagnosis of SCLC and reported different results. A wide range of sensitivity values were defined in SCLC, and this difference was thought to be explained by the use of different epitopic targets and different body fluids [16]. In a study using polyclonal anti-CgA antibodies by ELISA, sensitivity was shown to reach 39%, but CgA levels were found to increase with advanced disease stage, and a large number of patients in the study were reported to have advanced disease [31]. In the study of Børglum et al. with epitopes identified at the N-terminal end of CgA, the diagnosis was made with 58% sensitivity in SCLC with limited disease and with 100% sensitivity in extensive stage disease [32]. The low diagnostic susceptibility rates of 17%–42% in limited disease and 55%–67% in extensive disease reported in different trials using different epitopes demonstrate the effect of appropriate epitope selection on diagnostic sensitivity [30]. Although SCLC patients were mostly advanced stage, the CgA levels among the groups was not statistically significant in our study. This is because the present study included fewer SCLC patients. 

In our study, we hypothesized that SCCA1 could distinguish squamous cell carcinoma from PNET, but there was no significant difference among the groups in terms of SCCA1 values. In a study investigating the importance of SCCA1 in lung cancer and benign pulmonary diseases, it was shown that SCCA1 may also be increased in benign pulmonary diseases without squamous cell carcinoma (<20 ng/mL), but it did not exceed 40 ng/mL in any of the patients with benign lung cancer or squamous cell lung cancer. It has been reported that SCCA1 is not affected by age or sex but is associated with tumor progression and is detected at the highest levels in metastatic patients [21]. The nonsignificant difference in SCCA1 values between squamous cell carcinoma patients and PNET cases may be due to the study containing a low number of squamous cell lung cancer patients.

The present study has several limitations. First of all, age and sex were statistically significantly different in the 4 groups. The second limitation is that the number of subgroup patient participants was low, especially in the NSCLC group. The last limitation is that we did not follow patients for mortality, so we have no data about these markers and mortality in patients with PNET or malignancy.

In conclusion, ProGRP can distinguish PNET patients from NSCLC patients, and ProGRP with adjusted NSE values can differentiate the PNET group from the benign disease group. The ProGRP value is the only indicator that distinguishes the PNET group from the other 3 groups. After this study, our proposal for future studies is to investigate the long-term effect of ProGRP on mortality in PNET patients.

## Acknowledgment

This study was funded by the Scientific and Technological Research Council of Turkey (TÜBİTAK) with project number 115S013, under the scope of the 3001 project.
